# Brain-derived neurotrophic factor gene-modified bone marrow mesenchymal stem cells

**DOI:** 10.3892/etm.2014.2113

**Published:** 2014-12-08

**Authors:** ZHONG-MIN HAN, HE-MEI HUANG, FEI-FEI WANG

**Affiliations:** Department of Medical Technology, Zhengzhou Railway Vocational and Technical College, Zhengzhou, Henan 450052, P.R. China

**Keywords:** mesenchymal stem cells, brain-derived neurotrophic factor, differentiation, neuron-like cells

## Abstract

The present study aimed to investigate the effects of human brain-derived neurotrophic factor (hBDNF) on the differentiation of bone marrow mesenchymal stem cells (MSCs) into neuron-like cells. Lentiviral vectors carrying the hBDNF gene were used to modify the bone marrow stromal cells (BMSCs) of Sprague-Dawley (SD) rats. The rat BMSCs were isolated, cultured and identified. A lentivirus bearing hBDNF and enhanced green fluorescent protein (eGFP) genes was subcultured and used to infect the SD rat BMSCs. The expression of eGFP was observed under a fluorescence microscope to determine the infection rate and growth of the transfected cells. Methylthiazolyldiphenyl-tetrazolium bromide (MTT) was used to detect the proliferation rate of cells following transfection. Reverse transcription quantitative polymerase chain reaction (RT-qPCR) and western blot analysis were used to detect the expression levels of hBDNF. Differentiation of neuron-like cells was induced *in vitro* and the differentiation rate of the induced neural-like cells was compared with that in control groups and analyzed statistically. In the cultured cells, flow cytometry demonstrated positive expression of cluster of differentiation (CD)90 and CD44, and negative expression of CD34 and CD45. The proliferation rate of the rat BMSCs increased following gene transfection. The expression of hBDNF-eGFP was detected in the BMSCs of the experimental group. The differentiation rate of hBDNF-modified cells into neuron-like cells in the experimental group was higher compared with that in empty plasmid and untransfected negative control groups. The difference was statistically significant (P<0.05). Thus, BDNF gene transfection is able to promote the differentiation of BMSCs into neuron-like cells. BDNF may play an important role in the differentiation of MSCs into neuron-like cells.

## Introduction

Spinal cord injury (SCI) is a serious threat to the health and quality of life of those affected and its treatment has become a global issue. Brain-derived neurotrophic factor (BDNF) is a nerve growth factor that been reported to play an important role in the growth, development, differentiation, maintenance and damage repair of several types of neurons in the central nervous system. It also induces axonal regeneration and promotes neural pathways (1–3). As a class of pluripotent stem cells, mesenchymal stem cells (MSCs) have been widely used in cell transplantation for the treatment of SCI. However, studies have revealed that the lack of secretion of neurotrophic factors at the site of injury in the spinal cord and inadequately inducing conditions in the microenvironment influence the survival rate of transplanted MSCs and their differentiation into neurons, resulting in unsatisfactory neurological recovery (4–6). Therefore, developing a procedure to maintain the long-term survival of MSCs and raise their differentiation rate into neurons has become an important issue for the treatment of SCI by MSC transplantation.

With the advance of genetic modification technology, targeted genes have been used to transfect transplanted stem cells and stably express the gene products, thereby enhancing the effect of cell and gene therapies by incorporating the features of the gene. In a previous study, BDNF gene-modified MSCs were shown to promote functional recovery and reduce infarct size in a middle cerebral artery occlusion model of SCI (7). In the present study, the effect of the BDNF gene on the survival rate of MSCs and the rate of their differentiation into neuron-like cells was observed in BDNF gene-transfected MSCs.

## Materials and methods

### Isolation, culture and identification of bone marrow stromal cells (BMSCs)

Eight-week-old Sprague-Dawley (SD) rats (male or female) were sacrificed by cervical dislocation. The femur and tibia marrow cavities of the rats were exposed under sterile conditions and flushed with D-Hank’s solution containing heparin (100 U/ml). The fluid was collected and the BMSCs were isolated by density gradient centrifugation and an adherent method. Briefly, 1×10^6^ nucleated cells were loaded onto 25 ml of 1.073 g/ml Percoll solution and centrifuged at 1,100 × g for 30 min at 20°C. Cells were collected from the upper layer and interface, diluted with 2 volumes of Dulbecco’s phosphate-buffered saline (PBS) and collected by centrifugation at 900 × g. The cells were cultured in 25-ml culture flasks at 37°C with 5% CO_2_ in low glucose Dulbecco’s modified Eagle’s medium (DMEM; Invitrogen Life Technologies, Carlsbad, CA, USA) containing 10% fetal bovine serum (FBS; Hyclone, Logan, UT, USA), which was changed for the first time after 48 h and subsequently changed once every three days. The cells were digested with 0.25% trypsin (when cell fusion reached 80–90%), with subculture at a ratio of 1:3 (0.25% trypsin:cells) in different tubes. BMSCs growing on glass coverslips were washed three times with PBS and fixed with 0.3 mol/l NaCl and 75% ethanol for 30 min. Rabbit anti-mouse cluster of differentiation (CD)34, CD44, CD45 and CD90 antibodies (BD Biosciences, Franklin Lakes, USA) were added at a ratio of 1:100 to the BMSCs, which were incubated at 37°C in an incubator for 1 h. After washing twice with PBS, FITC-labeled goat anti-rabbit IgG secondary antibody (Pierce, Rockford, IL, USA) was added and the BMSCs were incubated at room temperature for 1 h. An inverted fluorescence phase contrast microscope (DFM-60; Shanghai Caikon Optical Instrument Ltd., Shanghai, China) was used to analyze the images.

Fourth generation cells with good growing conditions were selected to prepare single cell suspensions with ~3×10^5^ cells/sample. Following centrifugation at 500 × g for 5 min, the cells were resuspended in 500 μl PBS. The resuspended cell suspension was transferred to a test tube for flow cytometric analysis and 5 μl CD34, CD44, CD45 and CD90 antibodies were added, respectively. The cells were incubated for 30 min at 4°C. A total of 500 μl PBS was added to the cells, which were centrifuged at 500 × g for 5 min and the supernatant discarded. Following the addition of 300 μl PBS and mixing, the cells were analyzed using a flow cytometer (BD FACSCanto^™^ II; BD Biosciences). The data was analyzed using CellQuest software (BD Biosciences). The present study was approved by the Institutional Animal Care and Use Commitee of Zhengzhou Railway Vocational and Technical College (Zhegzhou, China).

### Grouping and gene transfection

A lentiviral gene expression vector carrying hBDNF and enhanced green fluorescent protein (eGFP) genes was produced by Cyagen Biosciences Inc. (Guanghzou, China) and amplified from HT1080 cells (acquired from Shanghai Meixuan Biotechnology Co., Ltd, Shanghai, China).

The cells were divided into three groups: hBDNF and eGFP gene transfection group (group A), empty lentiviral vector (LV-EGFP-0101; Cyagen Biosciences Inc.) transfection group (group B) and untransfected group (group C). Third-generation cells were used to perform viral transfection with a multiplicity of infection (MOI) of 25. The solution was changed after 8 h and cultured at 37°C in a saturated humidity incubator with a CO_2_ volume fraction of 0.05. The infection efficiency and cell growth were observed under a fluorescence microscope (DFM-60; Shanghai Caikon Optical Instrument Ltd.). G418 was added to the screen when the transfected cells had been cultured for 48 h.

### Methylthiazolyldiphenyl-tetrazolium bromide (MTT) assay

Cells from each group were seeded into 96-well plates with 1×10^4^/ml cells per well with eight wells for each group. A total of 200 μl cell culturing medium was added to each well and 20 μl MTT was added after 24 h at room temperture. The cells were incubated at 37°C for 4 h and the culture supernatant was removed. A total of 150 μl dimethyl sulfoxide (DMSO) was added to each well, the plate was oscillated for 10 min and the absorbance (A) value of each well at a wavelength of 490 nm was measured using a microplate reader (2550 EIA reader; Bio-Rad, Hercules, CA, USA).

### Western blot analysis

The medium of the hBDNF and eGFP gene transfection (group A), empty lentiviral vector transfection (group B) and non-transfected (group C) cells was aspirated and a lysis buffer was added. The cells were lysed using ultrasound (JYD-900; Shanghai Credibility Instrument Co., Ltd, Shanghai, China). A BCA Protein assay kit (Pierce) was used to detect the protein concentrations. A total of 20 μg of the protein was processed by 12% sodium dodecyl sulfate (SDS)-polyacrylamide gel electrophoresis and electrically transferred to a polyvinylidene difluoride (PVDF) membrane. The membrane was sealed with 5% skimmed milk for 1 h, the mouse anti-human GFP primary antibody (Pierce) was added and the membrane was incubated at 4°C overnight. Following washing of the membrane with Tris-buffered saline and Tween 20 (TBST), goat anti-mouse IgG-HRP secondary antibody (Pierce) was added and the membrane was incubated at room temperature for 60 min. Following washing with TBST, the membrane was analyzed by enhanced chemiluminescence [ECL; Santa Cruz Biotechnology (Shanghai) Co., Ltd., Shanghai, China]. The murine monoclonal anti-β-actin antibody [Santa Cruz Biotechnology (Shanghai) Co., Ltd.]. acted as the internal reference.

### Cell differentiation

The sixth generation cells of groups A, B and C were used for subculture. The induction of differentiation into neurons was performed when the adherent cell fusion rate reached 80% (8–11). The culture medium was aspirated, and 100% FBS and a medium containing 10 ng/ml basic fibroblast growth factor (bFGF) and DMEM/F12 were added for pre-induction for 24 h. The culture medium was aspirated and the cells were washed three times with PBS. DMEM/F12 serum-free inducer containing 20 ml/l DMSO, l0 ng/ml bFGF, 100 μmol/l butylated hydroxyanisole (BHA), 10 μmol/l forskolin, 25 mmol/l KCl, 2 mmol/l malonic acid and 5 μg/ml insulin was added. Half of the inducing agent was replaced every 24 h for three days.

### Immunofluorescence

Following removal of the medium, the cells were washed with PBS and fixed for 20 min with 4% polyformaldehyde at room temperature. The cells were subsequently washed twice with PBS, subjected to permeabilization with 0.1% Triton-X 100/PBS at room temperature for 1 h, then washed with PBS three times, for 5 min each time. Fresh blocking solution was prepared, and the cells were blocked with 5 mg/ml FBS/PBS for 30 min at room temperature. Polyclonal rabbit anti-mouse antibody [anti-TUJ-1 antibody; 1:50; Santa Cruz Biotechnology (Shanghai) Co., Ltd.] in blocking buffer was added to the cells, which were incubated at room temperature for 2 h. The cells were subsequently washed with PBS three times, for 10 min each time. PBS was used to prepare Cy3 fluorescence-labeled goat anti-rabbit IgG secondary antibody [1:400; Santa Cruz Biotechnology (Shanghai) Co., Ltd.], which was added to the cells and incubated overnight at 4°C. After washing three times with PBS, for 10 min each time, the cover slips were incubated with 4′,6-diamidino-2-phenylindole (DAPI) for nuclear staining and mounted with anti-fade mounting medium. The slides were observed under a fluorescence microscope (DFM-60; Shanghai Caikon Optical Instrument Ltd.) and images were captured. A total of 300 cells from each group were randomly selected following differentiation for 24 h and TUJ-1-positive cells were counted.

### Statistical analysis

Results are expressed as mean ± standard deviation. SPSS statistical software, version 17.0 (SPSS, Inc., Chicago, IL, USA) was used to carry out the statistical analyses and the factorial analysis of variance (ANOVA) was calculated. ANOVA was used to analyze the comparisons between groups. P<0.05 was considered to indicate a statistically significant difference.

## Results

### Microscopic observation

The adherent growth of primary cultured cells was visible following culture for 24 h, and a small colony appeared after 72 h. The cells grew in a tubular shape after 7–9 days and were basically fused after ~2 weeks. Immunofluorescence revealed that CD44 expression was positive and expression of the other surface antigens was negative. Following transfection by the hBDNF and eGFP genes, the appearance of the cells did not change significantly compared with the normal passage of cells, with long spindle or polygonal adherent growth, although the proliferation rate accelerated rapidly. Under a fluorescence microscope, green fluorescence was visible after 12 h with a low extent and weak intensity. Cells emitted strong fluorescence after 48 h, demonstrating the distribution of the whole cells.

### Determination of MSC surface markers

The results of flow cytometry detection and Cell Quest software analysis revealed that the positive rate of MSC-specific surface markers was 99.67% for CD90 (+), 99.81% for CD44 (+), 0.48% for CD34 (−) and 0.03% for CD45 (−).

### MTT assay

As shown in Table I, the A values of the cells in each group were detected at 490 nm following an MTT assay. The A value in the gene transfection group was significantly different from that in the empty vector-transfected and non-transfection groups (P<0.01). No statistically significant difference was identified between the empty vector and the non-transfected groups (P>0.05).

### Western blot analysis

**Figure 1:**
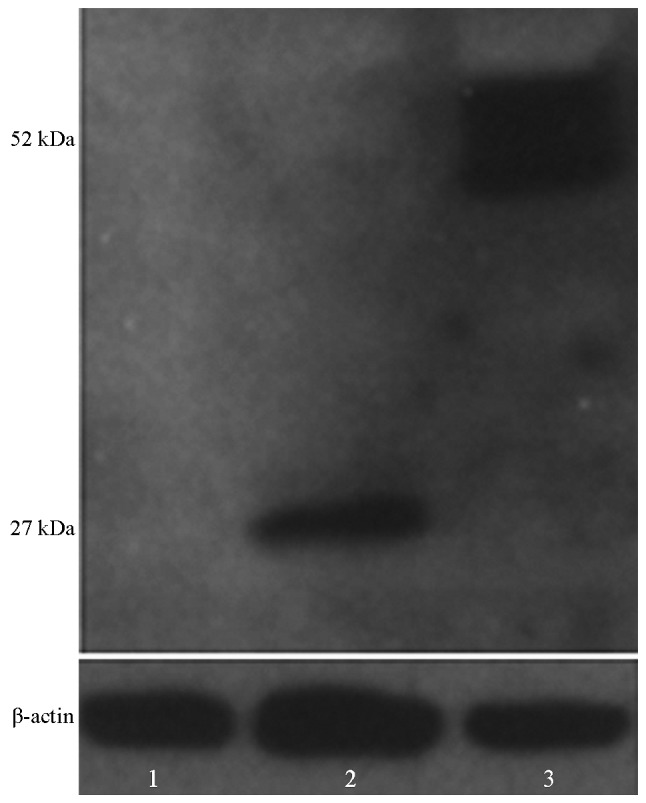
Expression of brain-derived neurotrophic factor **(**BNDF) in bone marrow stromal cells (BMSCs) detected by western blot analysis. Lane 1, control; lane 2, BMSCs transfected by empty vectors [only enhanced green fluorescent protein (eGFP)]; lane 3, BMCSs transfected by human brain-derived neurotrophic factor (hBDNF) and eGFP.

Stably expressing MSCs were obtained following BDNF and eGFP transfection. The expression of BDNF and GFP in the cells of the three groups was detected by western blotting using a GFP antibody. The results revealed that BDNF-GFP bands (~52 kDa) could be detected from the BDNF and eGFP-transfected cells via the GFP antibody. However, only GFP (~27 kDa) was detected in the empty vector-transfected cells (Fig. 1).

### Neuronal differentiation of MSC-like cells in vitro

Partial retraction of the cytoplasm to the nucleus occurred in the three MSC groups following the addition of the inducer. In addition, the cell bodies were reduced in size, irregular or rounded in shape, with three-dimensional, surrounding strong refraction. Frequently, several short projections and a longer projection were observed. Neuron-like cells exhibited a typical bipolar, multi-polar and tapered shape with a strong refraction. The neuron-specific marker TUJ-1 was used to stain the induced MSCs. TUJ-1 positive cells were detected in the three groups following the addition of the inducing agent. By comparing the TUJ-1-positive rate of the BDNF-transfected group with those of the empty plasmid and non-transfected negative control groups, it was demonstrated that the TUJ-1 positive rate in the experimental BDNF-transfected group was higher compared with that in the empty plasmid-transfected group (71.11±4.72 vs. 56.67±6.89%; P<0.05). The difference between the empty vector-transfected and negative control groups was not statistically significant (56.67±6.89 vs. 57.36±2.41%; P>0.05).

## Discussion

SCI is a serious central nervous system trauma. Although conventional treatment, including surgical intervention, drug treatment and postoperative rehabilitation training, following SCI is effective to an extent, limitations remain in the functional recovery of the spinal cord. In recent years, with the development of stem cell technology, the prospects for SCI treatment have improved through alternative neuronal cell transplantation technologies. Stem cells have a pluripotent ability and may differentiate into neurons under certain conditions, which have a significant recovery effect on neurological dysfunction caused by SCI. A number of sources of stem cells are available for the repair of SCI, including embryonic stem cells, neural stem cells, MSCs and cord blood stem cells. Due to certain characteristics of stem cells, including their ready availability, no transplant rejection occurs and directed differentiation of neuronal cells occurs. MSCs may be used as seed cells for wide application in the study of cell transplantation in the treatment of SCI (12).

However, due to local tissue hemorrhage, edema and cell necrosis following SCI, secondary ischemic changes and tissue cavity formation occur gradually at the site of injury. The damaged area lacks differentiation-inducing conditions, including neurotrophic factors, that are essential for the seed cells to differentiate into neurons. These factors result in low survival rates following MSC transplantation and lack of differentiation into neurons, which affects nerve function recovery in the treatment of SCI by cell transplantation (7,13). Previous studies have demonstrated that MSCs differentiate into neuron-like cells, which is associated with expression of the tyrosine kinase (Trk)B receptor (14–16). TrkB is a BDNF receptor for the cellular transmembrane protein. The binding of BDNF to TrkB receptors on the MSC cell membrane activates a series of cell bioreactors to promote the development of undifferentiated MSCs into mature neurons. BDNF is a member of the neurotrophic factor family that consists of α, β and γ subunits involved in the regulation of growth and differentiation of neurons in the central nervous system (8). Accordingly, gene modification technology was used in the current study to transfect exogenous BDNF gene into the MSCs. The results revealed that the expression of cell surface markers CD90 (+), CD44 (+), CD34 (−) and CD45 (−) by the cells was in line with that expected for MSCs, confirming that the cultured cells were MSCs from bone marrow and not hematopoietic cells from bone marrow.

At present, viral vectors including adenoviral, adeno-associated viral, retroviral and lentiviral vectors, are used for the transfer of targeted genes into seed cells, and each has both advantages and disadvantages. However, the application of non-viral vectors is relatively rare as their application in tissue engineering is challenging due to their low transfection efficiency (17,18).

In the current study, a BDNF-carrying lentiviral vector was used to infect rat MSCs, resulting in stable expression of the BDNF fusion protein by the MSCs. The successful transfection of BDNF into the MSCs and secretory expression of BDNF was confirmed by western blot analysis. The MSCs of the BDNF-transfected, empty vector-transfected and non-transfected negative control groups were induced to differentiate into neurons under similar conditions. All cells of the three groups expressed the neuronal specific antibody TUJ-1 following differentiation. The results revealed that the differentiation rate of the BDNF gene-modified MSCs into neural-like cells was significantly higher compared with that of the MSCs transfected with an empty vector and the negative control group (P<0.05), which was consistent with the results of Jouhilahti *et al* (11). This suggests that BDNF played an important role in neuron-like cell differentiation of the MSCs, although the exact mechanism remains unclear. In conclusion, the hBDNF gene carried by lentiviral vectors may promote the differentiation of MSCs into neuron-like cells *in vitro*. Further study is required to determine the function of the hBDNF gene *in vivo.*

## Figures and Tables

**Table I:** Effect of gene transfection on the proliferative activity of BMSCs (n=8 per group).

Group	A value
BMSCs transfected with hBDNF and eGFP	0.84±0.05
BMSCs transfected with empty vectors	0.42±0.04^a^
Non-transfected BMSCs	0.50±0.04^a^

Data are presented as mean ± standard deviation. BMSC, bone marrow stromal cell; hBDNF, human brain-derived neurotrophic factor; eGFP, green fluorescent protein.

a P<0.0, vs. the transfected group.
